# Hospital outbreak of carbapenem-resistant Enterobacterales associated with a *bla*_OXA-48_ plasmid carried mostly by *Escherichia coli* ST399

**DOI:** 10.1099/mgen.0.000675

**Published:** 2022-04-20

**Authors:** Alice Ledda, Martina Cummins, Liam P. Shaw, Elita Jauneikaite, Kevin Cole, Florent Lasalle, Deborah Barry, Jane Turton, Caryn Rosmarin, Sudy Anaraki, David Wareham, Nicole Stoesser, John Paul, Rohini Manuel, Benny P. Cherian, Xavier Didelot

**Affiliations:** ^1^​ Department of Infectious Disease Epidemiology, School of Public Health, Imperial College London, UK; ^2^​ Healthcare Associated Infections and Antimicrobial Resistance Division, National Infection Service, Public Health England, London, UK; ^3^​ Department of Microbiology and Infection Control, Barts Health NHS Trust, London, UK; ^4^​ Department of Zoology, University of Oxford, Oxford, UK; ^5^​ NHIR Health Protection Research Unit in Healthcare Associated Infections and Antimicrobial Resistance, Department of Infectious disease, Imperial College London, Hammersmith Campus, London, UK; ^6^​ Public Health England, London, UK; ^7^​ Microbes and Pathogens Programme, Wellcome Sanger Institute, Wellcome Genome Campus, Hinxton, UK; ^8^​ North East and North Central London Health Protection Team, Public Health England, London, UK; ^9^​ Modernising Medical Microbiology, Nuffield Department of Clinical Medicine, University of Oxford, John Radcliffe Hospital, Oxford, UK; ^10^​ Brighton and Sussex Medical school, Department of Global health and Infection, University of Sussex, Falmer, Brighton, UK; ^11^​ Public Health Laboratory London, National Infection Service, Public Health England, London, UK; ^12^​ School of Life Sciences and Department of Statistics, University of Warwick, Coventry, UK

**Keywords:** carbapenem resistance, enterobacterales, *E. coli*, conjugation rate, OXA-48, plasmid

## Abstract

A hospital outbreak of carbapenem-resistant Enterobacterales was detected by routine surveillance. Whole genome sequencing and subsequent analysis revealed a conserved promiscuous *bla*_OXA-48_ carrying plasmid as the defining factor within this outbreak. Four different species of Enterobacterales were involved in the outbreak. *Escherichia coli* ST399 accounted for 35 of all the 55 isolates. Comparative genomics analysis using publicly available *E. coli* ST399 genomes showed that the outbreak *E. coli* ST399 isolates formed a unique clade. We developed a mathematical model of pOXA-48-like plasmid transmission between host lineages and used it to estimate its conjugation rate, giving a lower bound of 0.23 conjugation events per lineage per year. Our analysis suggests that co-evolution between the pOXA-48-like plasmid and *E. coli* ST399 could have played a role in the outbreak. This is the first study to report carbapenem-resistant *E. coli* ST399 carrying blaOXA-48 as the main cause of a plasmid-borne outbreak within a hospital setting. Our findings suggest complementary roles for both plasmid conjugation and clonal expansion in the emergence of this outbreak.

## Data Summary

**Impact Statement**

Plasmids are autonomous genetic elements that can spread through neighbour bacteria, often belonging to different species. Thanks to recent advances in genomics analysis we can now study plasmids dynamics using WGS data. Here we present the analysis of a carbapenem resistance gene outbreak in a hospital ward. We find that the main driver of the outbreak is a very conserved conjugative plasmid, where the resistance gene is harboured. We also identified that the main bacterial host for the plasmid in this outbreak is an *E. coli* and not a *K. pneumoniae *. Further modelling analysis allows us to better understand the dynamic of the outbreak and make an estimation of the plasmid’s conjugation rate. This is an innovative approach to the study of plasmid outbreaks.

Sequencing data are available on ENA (study accession number PRJEB39204).

The awk scripts to transform the bam files into fastq and to run MUMmer and LASTZ are available on the github webpage https://github.com/aliceLedda/MgenOXA48/.

## Introduction

Horizontal gene transfer (HGT) is a key feature of bacterial adaptation [1]. HGT of genes from neighbour organisms helps bacteria to adjust to environmental change. Acquisition of antimicrobial resistance (AMR) through HGT represents a serious threat to public health [2]. HGT allows the spread of AMR genes within a single generation of bacteria. Bacterial host sensitivity for HGT may be narrow, with transfer limited to a single species, or broad, with spread of resistance genes between multiple species. Horizontal transfer of gene clusters may propagate multidrug-resistant (MDR) strains.

Plasmids are vectors of HGT, found ubiquitously in bacterial species [3, 4]. Conjugative plasmids are able to transfer copies of themselves to neighbouring bacteria and have been described as carriers of specific AMR genes [3, 5, 6]. One example is carbapenemases: genes such as *bla*_OXA-48_ and *bla*_KPC_ can confer resistance to carbapenems, a class of beta-lactam antibiotics [7–9]. These antibiotics are widely used in clinical practice as they are active against extended-spectrum β-lactamases ESBL-producing Enterobacterales which are resistant to most beta-lactams [10, 11]. Acquisition by bacteria that also produce ESBL may render them resistant to all beta-lactams currently used for treatment [8, 10, 12]. Several classes of carbapenemase have been described, and genes are typically found on plasmids and other mobile genetic elements (MGEs) [8, 10–13].

Plasmid pOXA-48 is a typical example of a plasmid carrying a gene (*bla*_OXA-48_) conferring resistance to carbapenems [8, 12]. pOXA-48-like plasmids have been found in a wide range of Enterobacterales [7, 14]. This presents a public health challenge: once acquired, the plasmid can spread among multiple species of Enterobacterales present in the gut microbiota, including potential pathogens. In this paper, we present a genomic analysis of an outbreak of carbapenem-resistant Enterobacterales associated with a pOXA-48-like plasmid in a hospital ward in the United Kingdom. Previous studies have reported multi-species outbreaks of pOXA-48-like in hospital settings [7, 15–17], but have remained largely descriptive. Here, we introduce a mathematical model of plasmid transmission between different bacterial host belonging to different genotypes and species to obtain a lower bound for the number of conjugation events per year.

## Methods

### Outbreak description

The outbreak occurred on a vascular surgery ward within a new hospital build. Patients on the ward were typically diabetic patients with diabetic foot ulcers or patients with poor vasculature needing leg revascularization. The ward had 30 beds, consisting of ten side rooms and five open four-bed bays, all with en-suite facilities. In each bay, beds were a minimum of 3.6 metres from bed-centre to bed-centre in line with health technical memoranda (HTM). In May 2016, a patient known to be colonized by *bla*_OXA-48_ *E. coli* was inadvertently admitted onto an open bay, due to oversight of their infection status when allocating their bed. This patient was mobile and socialised with other inpatients before the error was identified. At this point, the ward team screened patients on the same bay, identifying positive cases, and then began screening patients from other bays, identifying further positive cases. Further screening of patients in side rooms who were presumed not to have interacted with the patient also identified positive cases.

An outbreak was declared in June 2016 and cases continued to be identified by screening for 18 months. Although active surveillance was not conducted in the hospital at large, based on routinely submitted diagnostic specimens the outbreak appeared to be ward-specific. Initial standard infection and prevention control measures failed due to long durations of colonisation and frequent admissions to the ward. Stricter measures were later implemented, including education of healthcare workers, extension of the screening programme using PCR testing, enhanced cleaning, and emptying the ward. In total, screening over this 18 month outbreak identified 134 patients testing positive for *bla*_OXA-48_. The majority were asymptomatically colonised, although three patients developed an infection. Patients largely came from the community, but most had other admissions to the same hospital in the previous year. This pattern is characteristic of patients with chronic vascular insufficiency.

All clinical isolates showing carbapenem resistance were archived for further investigation. Here we analyse a subset from the first ten months of the outbreak: 55 isolates from 48 patients from May 2016 to February 2017. The gene conferring carbapenem resistance was identified using standard *in vitro* methods as *bla*_OXA-48_ and presence of *bla*_OXA-48_ was confirmed *in silico* using whole genome sequencing (WGS) data.

### Bacterial isolates and genomic sequencing

Isolates were cultured from −80 °C stocks onto Columbia Blood Agar (Oxoid, Basingstoke, UK) and incubated aerobically overnight. Bacterial DNA was then extracted and sequenced according to a previously described protocol [18]. In short a combination of physical (FastPrep, MP Biomedicals, USA) and chemical (QuickGene DNA Tissue Kit S, Fujifilm, Japan) lysis was used to lyse the bacterial cells and elute DNA. The DNA concentration was measured using the PicoGreen dsDNA Assay Kit (Waltham, Massachusetts, US). Whole-genome sequencing was performed using an Illumina HiSeq2000 that generated 100 bp paired-end reads (San Diego, California, US). Sequencing data are available on ENA (study accession number PRJEB39204).

### Genomic analysis

The read assembly was performed with SPAdes v3.11 [19]. Bacterial species identification was obtained from assembled contigs using the Nullarbor (https://github.com/tseemann/nullarbor)[20] suite and confirmed using the online version of KmerFinder [21–23] available on the Centre for Genomic Epidemiology website (http://www.genomicepidemiology.org/)[24][24]. Multilocus sequence typing (MLST) was performed using MLST on the same website.

Initial plasmid *de novo* assembly was done using the option PlasmidSpades in SPAdes v3.11 [19]. Since a reference plasmid was available (GenBank accession JN626286) [25], on-reference assembly was attempted using three different methods: lastZ [26], mummer [27] and samtools/bcftools [28, 29]. The final reference-based mapping was done using mummer [27].

For comparison to the existing diversity of pOXA-48-like plasmids, we searched PLSDB (v.2020_11_19) using the reference plasmid JN626286 with a minimum identity of 0.99 and a maximum p-value of 0.1, which identified 76 published plasmid sequences. We subsetted these further to those between 60–65 kbp in length (*n*=51). We compared these plasmids to each other and to the outbreak plasmids using mash dist (v2.0) with default parameters, then constructed a neighbour-joining tree from the resulting matrix of pairwise distance with *nj* in ape (v5.4–1) in R[30, 31,3]. This tree was midpoint-rooted and plotted using ggtree (v2.4.1).

Reference-based mapping approach was also used for *E. coli* chromosome assembly using samtools/bcftools [28, 29]. ECONIH4 [32] was used as a reference genome for all *E. coli* isolates in this study. The plasmid gene synteny map was made using an in-house pipeline. The annotated sequence of the reference pOXA-48 plasmid (GenBank record JN626286, identical to RefSeq record NC_019154) [8] was downloaded from GenBank (https://www.ncbi.nlm.nih.gov/genbank/) [25]. A customised blast database containing all the samples was created using the ‘*makeblastdb*’ application of the command line blast [33]. Each gene annotated in the reference plasmid was then blasted against this in-house database. Gene synteny was reconstructed using in-house programmes and plotted using the genoplotR package (http://genoplotr.r-forge.r-project.org/) [34] in R. A plasmid phylogenetic tree was built using the alignment resulting from reference based mapping using pOXA-48 as reference [8]. The tree was built using PhyML [35] in the analysis suite SeaView [36]. We used GTR model [37, 38] with default PhyML options. Nucleotide variants in the plasmid sequence were identified using the *seg.sites* command in the ape R package (https://CRAN.R-project.org/package=ape). ClonalFrameML [39] with default options was used to infer the plasmid tree.

For the comparative analysis of ST399 *E. coli* isolates, all available sequences of *E. coli* ST399 were downloaded from EnteroBase (http://enterobase.warwick.ac.uk/) [40]. These reads were processed following the same analysis pipeline as described for outbreak isolates.

### Expected number of mutations in pOXA-48-like plasmid

From standard population genetics theory, the number of mutations in a DNA sequence of length L during a time interval ∆t is expected to be $N_{mut}=\mu L\Delta t$ [41]. We assume that the *E. coli* mutation rate is $2.2610^{-7}$ per site per year based on previous estimates in [42]. This is a reliable estimation as it is not derived from experiments in a lab setting but from persistence in a household setting. pOXA-48 length is 61881 bp [8]. *E. coli* generation time being of the order of a few hours, we approximated seven generations a day [8, 43]. During the 15 years elapsed from the sampling of the reference pOXA-48 in 2001 and the sampling reported in this study in 2016, some 40000 generations have passed [43].

Therefore the expected number of mutations in a pOXA-48-like plasmid is 0.209 (±0.44) or ≦2 mutations at 95 % confidence interval. This estimate assumes the pOXA-48 reference as a direct ancestor to the present outbreak, and defines a lower limit to the number of expected mutations.

### Neutral model of inter-bacterial-host conjugation

We considered a neutral model of plasmid conjugation among bacterial hosts. The model is based on a Markov process tracking the probability that *a single plasmid lineage* switches between different host strains. The output of the model is the probability of observing a single plasmid lineage in a given host/strain, which corresponds to the expected host frequency. Conjugation is modelled as a homogeneous Markov process between any two bacterial hosts. In developing the model we rely on four realistic, albeit somewhat restrictive, assumptions: (i) that the taxonomic classification of the hosts is neutral, (ii) that the host populations have all similar abundances, (iii) that the epidemics started with a single host and (iv) the number of potential hosts is finite and equal to the ones in the samples at hand. Each different ST within each observed species is treated as a different host and STs that belong to the same species or to different species are considered equally different. Therefore we have a total of 21 different potential hosts, with the plasmid conjugating indifferently between any two bacterial hosts.

The Markov process generator matrix M describing the instantaneous conjugation probability between different hosts in the Markov chain is therefore a 21×21 isotropic matrix. The host neutrality assumption (i) implies that the matrix depends on a single parameter, the conjugation rate *r*. More specifically, including the requirement that each row sums to 0, M has values −r on the diagonal elements and r/20 off-diagonal. The assumption of all the hosts being neutral combined with the assumption that the epidemics started with a single host implies that the most likely most recent common ancestor (MRCA) of the outbreak was a pOXA-48 in *E. coli* ST399 strain. Therefore, the Maximum Likelihood initial state distribution of the Markov process is a vector with one in the element *E. coli* ST399 and 0 in all the other elements corresponding to the other hosts.

The dynamics of a Markov process with a finite number of states is fully determined by its generator matrix. Which means that in this situation, once we know the generator matrix and the initial state, we know at any given moment the distribution of hosts where the plasmid conjugated. Given the initial distribution π(0)=π(MRCA) at *t_0_*, i.e. at the time of the MRCA, the distribution at any following time *t* is given by $\pi (t)=\pi_{o}e^{Mt}$ , with M the matrix shown above. The probability that over all the time passed since the most recent common ancestor the plasmid never conjugated out of *E. coli* ST399 is $e^{-rt}$ with r being the conjugation rate. Since the plasmid has only two options, namely either it conjugates out or it does not, the probability that, during the same time, it conjugated out is $1-e^{-rt}$ .

The probability that the plasmid is found in *E. coli* ST399 at a time *t* after the time of the most recent common ancestor *t_0_* is the projection on *E. coli* ST399 of the exponential of matrix M:

$P_{conj}\left(t\right)=P_{ST399}\left(t\right)={\left[e^{Mt}\right]}_{ST399,ST399}=\frac{1}{21}+\frac{20}{21}e^{\frac{-21}{20}r\left(t-t_{0}\right)}$ (1)

as $\pi_{ST399}\left(0\right)=1$ . The first term is time independent and corresponds to the stationary asymptotic state of the system, while the second term is time dependent, decreasing with time from *t_0_*.

In the outbreak at hand, frequentist the probability that the plasmid conjugates out of *E. coli* ST399 is 0.36 since *E. coli* ST399 is not the host in 20 of the 55 samples in this study. Equating this probability to $e^{-rt}$ , we get rt=ln0.36 . Using this point estimate, we are able to estimate the probability that the plasmid is found in *E. coli* ST399 as a result of it conjugating back into it from another bacterial host in which it has previously conjugated during the time elapsed from the MRCA. This probability is the probability of finding pOXA-48 in *E. coli* ST399 (Equation 1) minus the probability that it never conjugated out $e^{-rT}$ . The result is ≃ 0.033.

So there is around 3 % of chance that we find pOXA-48-like in a *E. coli* ST399 as a result of its conjugation from another bacterial host, and 97 % probability that it comes either from vertical inheritance or conjugation from another *E. coli* ST399.

### Estimation of pOXA-48 conjugation rate

The evolutionary model described in ‘Neutral model of inter-bacterial-host conjugation’ can be seen as a special case of a Wright-Fisher model [44, 45] with symmetric discrete traits. In the Wright-Fisher model the alleles of the gene found in the next generation are a random sample of the alleles in the previous generation plus new mutations. In this case the bacterial hosts found in the next generation are a sample of the ones found in the previous one, plus new hosts. This mathematical parallel allows us to apply a coalescent approach to determine the conjugation rate in our sample. The coalescent approach consists in applying a Kingman coalescent [46] with conjugation instead of mutations happening at a constant rate along the tree branches. Conjugation changes the host from *E. coli* ST399, which we have seen is the initial host of the epidemics, to any other host. We make use of the infinite sites model approximation as it does not clash with the assumption we made of having a finite number of hosts. The non-ST399 *E. coli* hosts in our sample are more than 1/3, and, more importantly, they are all different from one another. This gives an effective decreasing probability that the plasmid conjugates twice in the same non-ST399 *E. coli* host (*P*<1/55~0.01), which justifies using the infinite sites model approximation [45, 46].

The probability of a conjugation event per unit time is $r_{conj}dt$ . Integrating over the tree height, the conjugation probability is $p_{conj}=1-e^{r_{conj}T_{MRCA}}$ . On the other end $p_{conj}$ computed in a frequentist manner from our sample is 20/55=0.36, as there are 20 non-* E. coli* ST399 hosts.

A dated phylogeny of the *E. coli* in the sample was obtained using BactDating [47]. It was used to infer the time since the MRCA (TMRCA) of the *E. coli* ST399 in the sample using a mutation rate per site per year of 2.26×10^−7^ [42] for the same reasons stated above. The estimation puts TMRCA around September 2014, so around 2 years before the onset of this outbreak. Feeding all these numbers into the equation $p_{conj}=1-e^{r_{conj}T_{MRCA}}$ we get $0.36=1-e^{r_{conj}2\left(years\right)}.$ Using this point estimate, we infer a conjugation rate of $r_{conj}≅0.23\left(\pm 0.09\right)$ per lineage per year.

## Results

### Outbreak overview

**Fig. 1.**
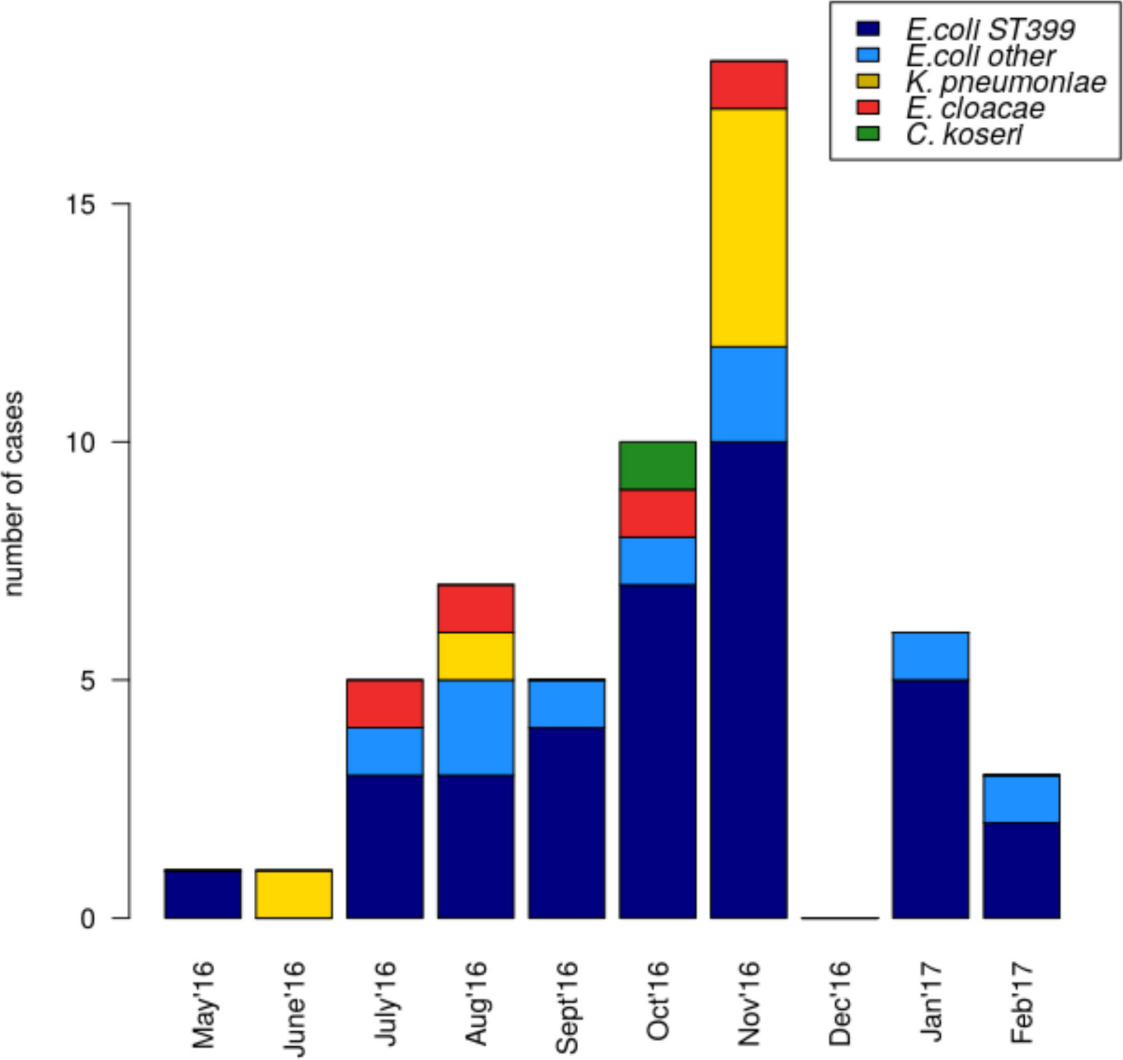
Progression of the *bla*_OXA-48_ outbreak, the main bacterial isolates and MLST types involved are highlighted in different colours: *E. coli* ST399 in dark blue, all other *E. coli* types in light blue, *Klebsiella pneumoniae* in yellow, *Enterobacter Cloacae* in red and *Citrobacter koseri* in green.

An eighteen-month outbreak of carbapenem-resistant Enterobacterales occurred on a vascular surgery ward within a new hospital building, with the first known case detected in May 2016 (see Methods). A total of 55 carbapenem-resistant isolates were cultured from 48 unique patients within the ten months of the outbreak (May 2016 to February 2017). From both *in vitro* assays and subsequent whole genome sequencing, all isolates were confirmed to carry the *bla*_OXA-48_ gene on a pOXA-48-like plasmid. Four different species of Enterobacterales were identified in the outbreak (Fig. 1). Most isolates were *Escherichia coli* (*n*=43) in blue in Fig. 1, with *Klebsiella pneumoniae* (*n*=7), *Enterobacter cloacae* (*n*=4) and *Citrobacter koseri* (*n*=1) also present (respectively in yellow, red and green in the Fig. 1).

Thirty-five of the 43 *E. coli* isolates (81%) belonged to ST399 based on the Achtman multi-locus sequence typing (MLST) scheme of *E. coli* [48, 49]. Eight other *E. coli* strains belonged to different STs, including two novel STs (Table S1, available in the online version of this article). *E. coli* ST399 has previously been described as a carrier of the pOXA-48-like plasmid, although it was not a main bacterial host carrying this plasmid in the reported study as only two instances of carriage were reported [50]. Additionally, in our study none of the *bla*_OXA-48_ -positive *E. coli* isolates were ST38, which was previously found associated with *bla*_OXA-48_ but not with pOXA-48-like plasmid carrriage [51]. None of the seven *K. pneumoniae* isolates were ST14 or ST10, previously found associated with pOXA-48-like carriage [50], and only one of the four *E. cloacae* isolates belonged to a previously described ST (Table S1).

The index patient case carried an *E. coli* isolate belonging to ST399 sampled at the end of May 2016, and second case carried *K. pneumoniae* isolate sampled in mid-June 2016 (Fig. 1). Since June 2016, the number of cases found positive for carrying carbapenem-resistant bacteria had increased until ward closure to new patient admissions in December 2016 due to continuous increased number of patients affected by *bla*_OXA-48_ -positive Enterobacteriaceae. The ward was reopened in January 2017, and only carbapenem-resistant *E. coli* cases were detected since.

### Highly conserved pOXA-48-like plasmid

**Fig. 2.**
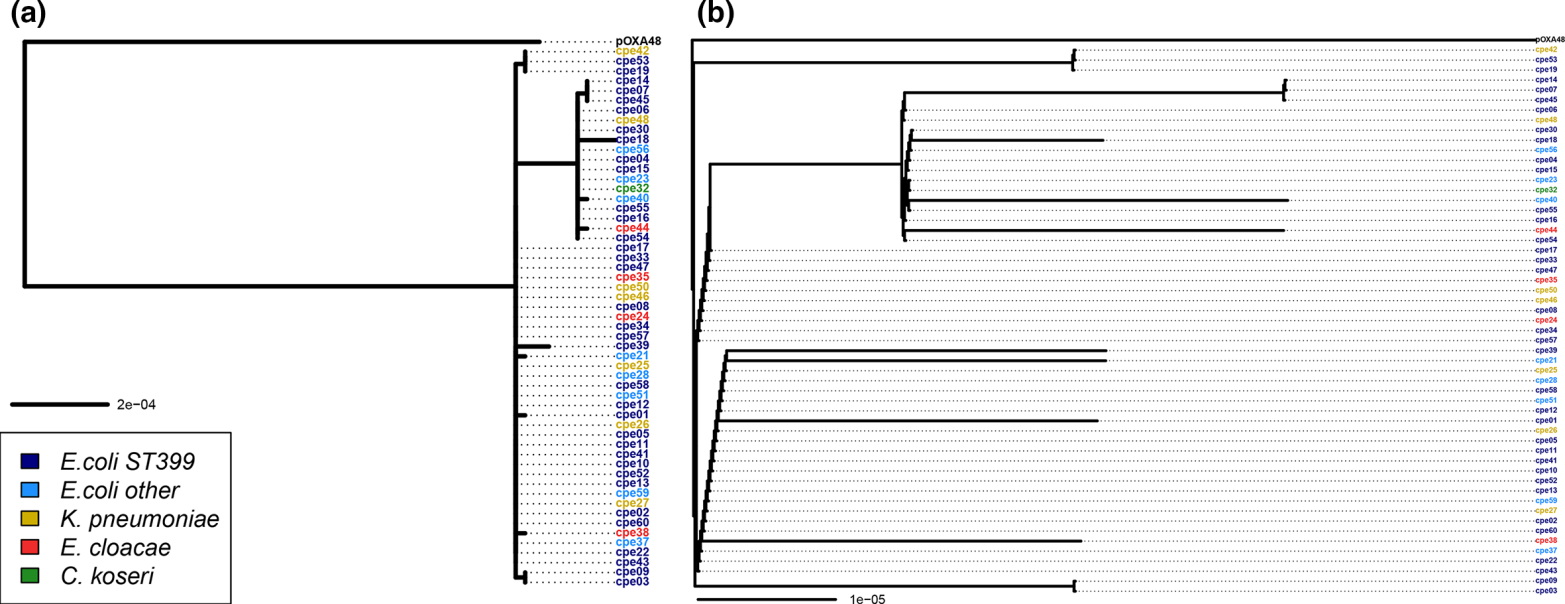
Phylogenetic analysis of the pOXA-48 plasmids. (a) Phylogenetic tree of all the pOXA-48 sequences estimated with PhyML under the GTR model (b) Clonal genealogy estimated by ClonalFrameML.

All of the 55 isolates were confirmed *in silico* to be carrying the *bla*_OXA-48_ allele, as previously described (no *bla*_OXA-181_ or *bla*_OXA-163_, which have a similar phenotype, were found) [11, 52]. The plasmid pOXA-48 is a 61881 bp plasmid belonging to the L/M incompatibility group (IncL/M), that mainly carries replication and conjugation machinery along with the *bla*_OXA-48_ gene [53]. Thus, it was no surprise when all *bla*_OXA-48_ genes identified in our strains were present on a pOXA-48-like plasmid. No specific insertions or deletions (compared to previously described pOXA-48 reference (NCBI GenBank accession JN626286 [8]) were found that could be uniquely attributed only to our outbreak strains. The genomic content of the pOXA-48 plasmid in our isolates was highly conserved. Genomic comparison to previously published pOXA-48-like plasmids (*n*=51) showed that our outbreak plasmids were largely distinct (Fig. S1a). The closest publicly available plasmids were from environmental samples in France: *K. pneumoniae* isolated from a water dam (NZ_MK249856), *E. cloacae* isolated from a vegetable (NZ_MK249855), and *E. coli* from a wild bird (NZ_MK249858). Host species were intermixed in the tree (Fig. S1b). An alignment of all pOXA-48 plasmids in this study (*n*=55), were used to construct a phylogenetic tree in which the reference pOXA-48 was used as an outgroup (Fig. 2a). Plasmids from different bacterial species were intermixed within the phylogeny tree (Fig. 2a), indicating that the outbreak was not caused by expansion of a single clone of one of the bacterial species identified in the study.

### Recombination analysis

ClonalFrameML was used to identify the recombination events among the studied plasmids (Fig. 2b). The overall tree topology remained unchanged once recombined segments of the plasmid were removed (Fig. 2), and reference pOXA-48 plasmid remained clearly separated from all other plasmids in the phylogeny tree (Fig. 2b).

**Fig. 3.**
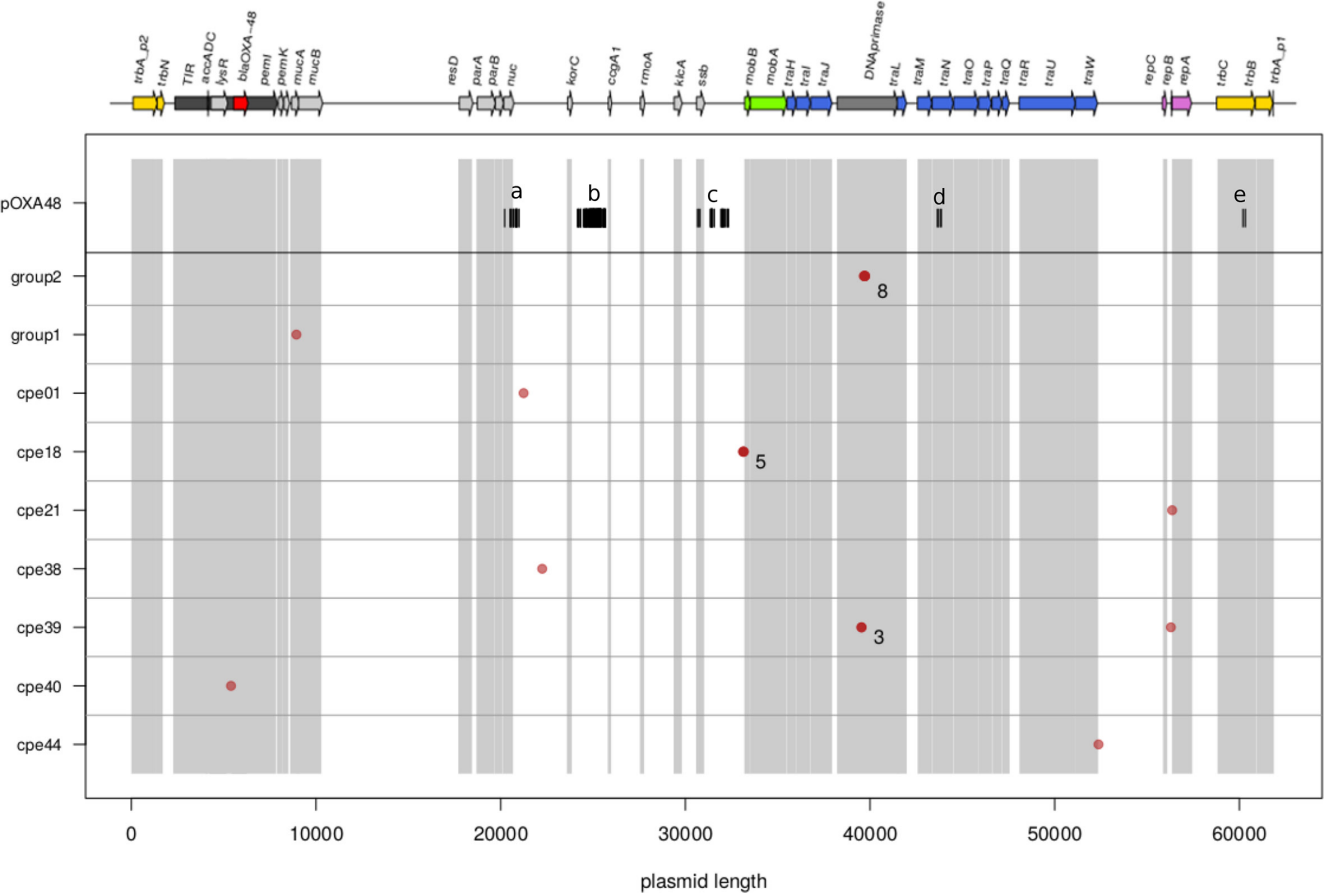
Genomic distribution of polymorphisms. On the x axis the plasmid length, the coding regions are highlighted by shaded areas and the plasmid annotation is given on the top. On the y axis the isolates where the polymorphisms are found. The polymorphisms found in the present dataset are shown by red dots while the ones characterising pOXA-48 are given by black lines. When more than one polymorphism is present in a restricted region the number is given next to the symbol. For details of the polymorphisms characterising pOXA-48 see text and Table 1. * Group one plasmids: cpe53, cpe42, cpe07, cpe14, cpe45, cpe19. * Group two plasmids: cpe44, cpe48, cpe54, cpe55, cpe07, cpe32, cpe14, cpe23, cpe18, cpe30, cpe56, cpe04, cpe15, cpe45, cpe40, cpe16.

To understand better the evolutionary history of the plasmids identified during the outbreak, all nucleotide differences between any pair of the 55 plasmids were identified (including the reference pOXA-48). The identified mutations are shown in Fig. 3. A total of 155 nucleotide variants were found, 129 of which distinguished the pOXA-48 reference from all other plasmids in the dataset (Fig. 3 upper row).

In non-recombining sequences with weak mutational biases, neutral mutations are expected to be distributed almost uniformly along the sequence. This is clearly not the case of the variants which distinguished outbreak plasmid sequences from the pOXA-48 reference, which had mutations localised in five clusters (identified with letters a-e in the upper row of Fig. 3 and in Table 1). This mutation pattern can be interpreted as resulting from several independent homologous recombination events, although other interpretations are possible. Four out of five of these putative events (a, c, d and e in the upper row of Fig. 3 and in Table 1) are located in coding regions, and one (b in the upper row of Fig. 3 and in Table 1) is located in an intergenic region. Most of the identified variants (114 out of 129) lie in intergenic regions, with the majority of these variants concentrated in the second recombinant locus (locus b in Fig. 3, Table 1). Of the 15 mutations located in coding regions, ten are synonymous and five are non-synonymous. The five non-synonymous mutations are located in three genes (*nuc, ssb* and *traN*) involved in pilus formation and single stranded DNA mobilization, so important for the plasmid’s conjugation effectiveness. The fact that all of them reside in the first part of each gene points to them being functional, although describing their function goes beyond the scope of this paper.

**Table 1.** Nucleotide differences between the sampled plasmids and pOXA-48 (see also Fig. 3). The first column refers to the location of the region in Fig. 3, the second column gives the position of the two SNPs delimiting the putative recombinant region, the third column reports the number of SNPs in the region, the fourth the name of any gene located in the region and the last column gives more detail on the SNPs: how many are located in intergenic regions, how many are non-synonymous and for the synonymous the mutation(s) with respect to the reference pOXA-48 are given

	Recombinant region (bp)	no. of SNPs	Gene	Intergenic	Syn	non-syn
*a*	20514–20 961	11	*nuc*	5	4	T31A, G174D
*b*	24206–25674	95	–	95	–	–
*c*	30661–32 310	17	*ssb*	14	2	R27G
*d*	43636–43 837	4	*traN*	–	2	Q89R, T156G
*e*	60324–61 717	2	*trbC*	–	2	–

Overall, the pOXA-48 plasmid gene content was highly conserved in all investigated pOXA-48-like isolates. No single nucleotide variant common to the whole outbreak dataset and different from the reference plasmid was observed outside of the putative recombination events, suggesting that no nucleotide substitution occurred during the diversification of the plasmids in our sample. Considering the length of the plasmid and the time elapsed since the sampling of the reference pOXA-48 plasmid we would have expected to see very few, if any, nucleotide substitutions (see Methods section ‘Expected number of mutations’). Similarly, we observed no structural variants such as insertion/deletion of insertion sequences, transposon or any other genetic material.

### Outbreak dynamics and estimation of inter-host conjugation rate

All the hosts except *E. coli* ST399 appear only once in our dataset. So the host frequency spectrum is composed of singletons, except for *E. coli* ST399, which was also the first host identified in the outbreak. Two evolutionary scenarios are consistent with this pattern: (i) *E. coli* ST399, in combination with pOXA-48, had an evolutionary advantage compared to other host-plasmid combinations (‘co-evolution' scenario), or (ii) *E. coli* ST399-pOXA-48-like association resulted from the recent rapid expansion of *E. coli* ST399 ('neutral association' scenario). The hypothesis of co-evolution of pOXA-48 with *E. coli* ST399 implies that the plasmid potentially has reduced fitness in other bacterial hosts. In such a scenario, the fact that we find the plasmid just once in all the other hosts (i.e. the other hosts appear as singletons in the host frequency spectrum) can be explained as resulting from very recent conjugation events out of *E. coli* ST399, with the resulting plasmid-host pairs not yet purged by purifying selection [54, 55]. In the ‘neutral association’ scenario, the rapid expansion of the plasmid population would generate a star-like genealogical tree for the plasmids. This could happen if for example there was a sudden increase in the carbapenem usage and the presence of the plasmid would confer a very high fitness to any of its hosts. In this scenario each plasmid-host lineage evolved and conjugated independently after the start of the outbreak [54].

To better understand the dynamic of this outbreak in a population genetics theoretical framework, we built a Markov model of plasmid transmission between hosts for the ‘neutral association’ scenario, which relies on the following assumptions: the epidemic started from a single host, there is no unsampled host population, and plasmids conjugate indifferently between any two bacterial hosts. For more details see Methods section ‘Neutral model of inter-bacterial-host conjugation’. This neutral model supports the idea that the founder host for this outbreak is indeed *E. coli* ST399: in fact, assuming neutral association between the plasmid and any of the hosts, the most abundant host today is most likely the original host of the plasmid (see Methods). The model predicts that plasmids found in *E. coli* ST399 have been either vertically inherited or obtained by intra-host conjugation from other ST399 *E. coli* strains, as conjugation from another host into *E. coli* ST399 was highly unlikely (see Methods section ‘Expected number of mutations’). The neutral model was then used to infer the underlying evolutionary scenario following a host-switching process under an ‘infinite-hosts’ conjugation model to draw conclusions on the evolutionary history of the outbreak based on the host frequency spectrum of pOXA-48 [54]. The inter-host conjugation rate under the assumption of the neutral association scenario is easily inferred. In this scenario, the plasmid tree would be approximately star-like and all non-ST399 *E. coli* hosts would result from independent conjugation events. All conjugation events would have occurred at random times between the present and the time of the most recent common ancestor (tMRCA) (see Methods section ‘Estimation of pOXA-48 conjugation rate’). In our dataset, BactDating [47] estimates that the most recent common ancestor of the *E. coli* ST399 tree is around late 2013 (data not shown). Using this information, we estimate a pOXA-48 conjugation rate of about 0.23 conjugation events per lineage per year.

### A case of co-evolution between plasmid and host?

The genomic data available could be explained by both the recent neutral expansion scenario or the co-evolution scenario or any scenario in between. Although we used the neutral scenario hypothesis to estimate a lower bound to the conjugation rate in this outbreak, the co-evolution hypothesis cannot be discarded. Therefore, the possibility of co-evolution between *E. coli* ST399 and the plasmid responsible for the outbreak was investigated in more detail. To provide context to our observations, we checked whether the studied outbreak might be part of a broader epidemic of *E. coli* ST399. We searched EnteroBase to obtain all publicly available *E. coli* ST399 (*n*=82) and these were then mapped to the *E. coli* ST399 reference genome and to the reference plasmid pOXA-48 (see Methods).

The search for the plasmid in the downloaded dataset yielded inconclusive results as no single full-length pOXA-48 plasmid was found. No *bla*_OXA-48_ gene was found to occur within the publicly available genomes of *E. coli* ST399 (*n*=79/82); only partial fragments of the plasmid that did not include the resistance gene *bla*_OXA-48_ were found in 20 of these genomes.

A phylogenetic tree of *E. coli* ST399 including the reference, the isolates included in the present study and the genomes from EnteroBase is shown in Fig. S2. All *E. coli* ST399 isolates from this study, except two, clustered in a single clade, pointing to an expanding outbreak of vertically inherited pOXA-48. The two isolates, separated from the main clade, were sequenced at the end of the sampling period: in January and February 2017. None of the other EnteroBase sequences formed clusters within the same clade, indicating the outbreak had a single main source associated with the hospital ward reported in this study.

## Discussion

In this paper we present the analysis of an outbreak caused by plasmid pOXA-48, carrying antimicrobial resistance genes, and detected by routine surveillance in a hospital ward. This study covers the first ten months of the 18 months long outbreak. The outbreak involved several Enterobacterales species and was characterised by a carbapenem resistance [13, 50]. Sequencing data revealed a *bla*_OXA-48_ gene carried in a highly conserved pOXA-48-like plasmid. The plasmid was very conserved, which aided in assembling the full-length sequences of the plasmid from Illumina short-read sequencing, without the need for long-read sequencing. Contrary to previously described pOXA-48 outbreaks [7, 12–14]; the main host in our study was *E. coli* not *Klebsiella pneumoniae.* More than half of the *E. coli* isolates from the outbreak were *E. coli* ST399, while the others were different STs. Previously, *E. coli* ST38 has been described as the main bacterial host in a *bla*_OXA-48_ outbreak, but the plasmid had been partially inserted in the chromosome, meaning that outbreak relied on the clonal expansion of the bacterial host and no plasmids were really involved [51].

To have a better understanding of the dynamics of this outbreak we used a mathematical modelling approach. First, we used a well-known population genetics formula to infer the expected number of mutations in pOXA-48 plasmid, which was found to be much lower than the number observed in the outbreak at hand. This result was a lower bound for the number of expected mutations as it relied on the assumption that pOXA-48 was a direct ancestor to the pOXA-48-like plasmids in this outbreak. The dating of the most recent common ancestor was made using tools developed for chromosomes; we know that plasmid and chromosome evolution are quite different, in particular plasmid evolution is less reliant on point mutation and more on conjugation/structural variation, but at the moment no tools explicitly developed for plasmid are available.

Secondly, we made a very simple neutral model of plasmid conjugation that described how the plasmid conjugates into different Enterobacterales hosts. The model relies on four very simple assumptions: all hosts are neutral, so for none of the hosts the association with the plasmid produces a fitness advantage (nor any of the hosts is advantageous for the plasmid) (i), all the hosts have a similar abundance in the reservoir (ii), that the epidemics started by the association of a plasmid with a single host (iii) and that the hosts that we found in this dataset were all the equally possible hosts for this plasmid in this outbreak (iv). Note that combining together the assumption that all the hosts are neutral and that the outbreak started from a single host implies that the most recent common ancestor for this outbreak is the most frequent host, that is *E. coli* ST399.

Describing conjugation in this outbreak as a homogeneous Markov process that fulfils these assumptions results in a very simple analytical system: there is only one conjugation rate between any two hosts and once we know the most recent common ancestor we are able to predict the state of the system at any subsequent time. This allows us to compute the probability that a plasmid that is originally hosted in *E. coli* ST399 conjugates into another host and then again into *E. coli* ST399. This probability is 0.033, which makes it a very unlikely event compared with the probability it never conjugates into another host (which is 0.64), thus supporting the idea that the plasmid is mainly inherited vertically in *E. coli* ST399. This supports the idea that *E.coli* ST399 with pOXA-48-like constitutes the vertical backbone of the outbreak while all the other hosts are resulting from horizontal transfer.

Finally, we used this model to build a model of conjugation through time inspired by the Kingman coalescent. The Kingman coalescent is typically used to describe the ancestral process for sequences evolving under the Wright-Fisher model with mutations. Here we use the plasmid’s phylogenetic tree to infer the conjugation rate using the model above as a description of the conjugation process.

Because of the plasmid length (~61 kbp) [8, 53] and relative brevity of the outbreak, not enough mutations accumulated to allow us to resolve the plasmid phylogenetic tree and ascertain the precise evolutionary scenario involved in the outbreak. It might be a ‘co-evolutionary scenario’ or a ‘neutral association scenario’. In the first case the association between pOXA-48 and *E. coli* ST399 is a favourable one and pOXA-48 in any other bacterial background is not as fit and eventually gets purged by natural selection. In the second scenario pOXA-48 underwent a recent expansion and all the conjugation events happened after the beginning of the outbreak, but no plasmid-host association has an increased fitness.

The first case, ‘co-evolutionary scenario’ is more difficult to model, as we would need to have an idea of the different fitness that the plasmid has in the different hosts, and this would add parameters to our estimation. In the ‘neutral association scenario’ case of a recent expansion with a star-like tree the plasmid conjugation rate is readily estimated. This rate is in fact a lower bound for the real conjugation rate in this specific outbreak. It might be a gross under-estimation of the conjugation rate if the plasmid is strongly adapted to *E. coli* ST399. In such a case, conjugation events would have occurred much more recently than estimated, by a factor given (in bacterial generations) by the inverse fitness advantage of *E. coli* ST399 compared to other hosts. The timescale of these plasmid transmission events could thus be much shorter than the time to the most recent common ancestor of all plasmids in this study, hence resulting in a much faster conjugation rate. So 0.23 per lineage per year is a lower bound to the real conjugation rate in our sample. This conjugation rate over time cannot be directly compared to *in vitro* experiments, which measure the conjugation frequency: the proportion of colonies infected over the total number of colonies during the course of an experiment. Nevertheless, such experiments confirm that pOXA-48 can be mobilized between species and genera with a high conjugation frequency [14, 51].

It is important to notice that our model does not make the assumption that the outbreak is limited to the specific hospital ward. The isolates involved are non-virulent and can spread unnoticed in absence of active surveillance. During this outbreak, active surveillance was carried out only in the involved ward. This specific ward is in constant exchange with the community, so the outbreak could be localised in the ward-community continuum. To better grapple with silent outbreaks like this one extensive active surveillance both in healthcare settings and in the community even in apparent absence of relevant outbreaks.

Surveillance combined with detailed analysis and modelling is of paramount importance for early detection and management of plasmid outbreaks. While surveillance is important in its own right, we have shown the value of whole-genome sequencing and subsequent mathematical modelling. We managed to identify the clonal part of the outbreak, which is the most important one to stop in order to end the outbreak overall. We have also estimated the conjugation rate, which is useful to infer how much spill-out to expect. Future analysis of surveillance data will be valuable, not only to detect plasmid outbreaks very early on but also to provide guides to the best way to stop those outbreaks.

## Supplementary Data

Supplementary material 1

Supplementary material 2
