# Supplementary material for: Hospital outbreak of carbapenem-resistant Enterobacterales associated with a bla OXA-48 plasmid carried mostly by Escherichia coli ST399
Source: Microb Genom. 2022 Apr 20;8(4):000675. doi: 10.1099/mgen.0.000675 (PMC9453065; doi:10.1099/mgen.0.000675)
Supplement: Supplementary material 1 [file mgen-8-0675-s001.pdf]

Hospital outbreak of carbapenem-resistant Enterobacterales associated with a *bla*$_{OXA-48}$ plasmid carried mostly by *Escherichia coli* ST399

Alice Ledda, Martina Cummins , Liam P. Shaw, Elita Jauneikaite, Kevin Cole, Florent Lasalle, Deborah Barry, Jane Turton, Caryn Rosmarin, Sudy Anaraki, David Wareham, Nicole Stoesser, John Paul, Rohini Manuel, Benny P Cherian , Xavier Didelot

Supplementary Material

Figure S1……………………………………………………………………………………1
Figure S2……………………………………………………………………………………2
Supplementary Table 1: Species Identification……………………………………………..3
Supplementary Table 1: mlst………………………………………………………………..6
Supplementary Table 1: Assembly…………………………………………………………..8
Supplementary Table 1: Sequencing Data…………………………………………………..12
Supplementary Table 1: Epidemiological Data……………………………………………..14

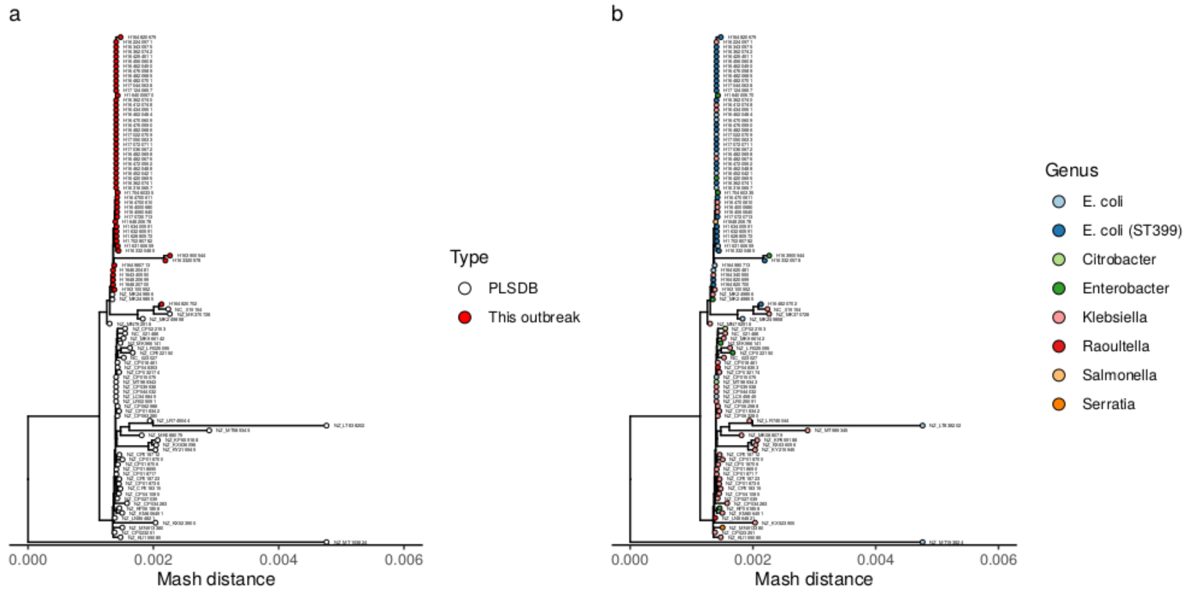

Figure S1: A neighbour-joining tree of alignment-free distances between published pOXA-48-like plasmids and those in this outbreak (see Methods). The tree is midpoint-rooted. (a) Plasmids coloured by involvement in this outbreak (red) or not (white). (b) Plasmids coloured by host genus.

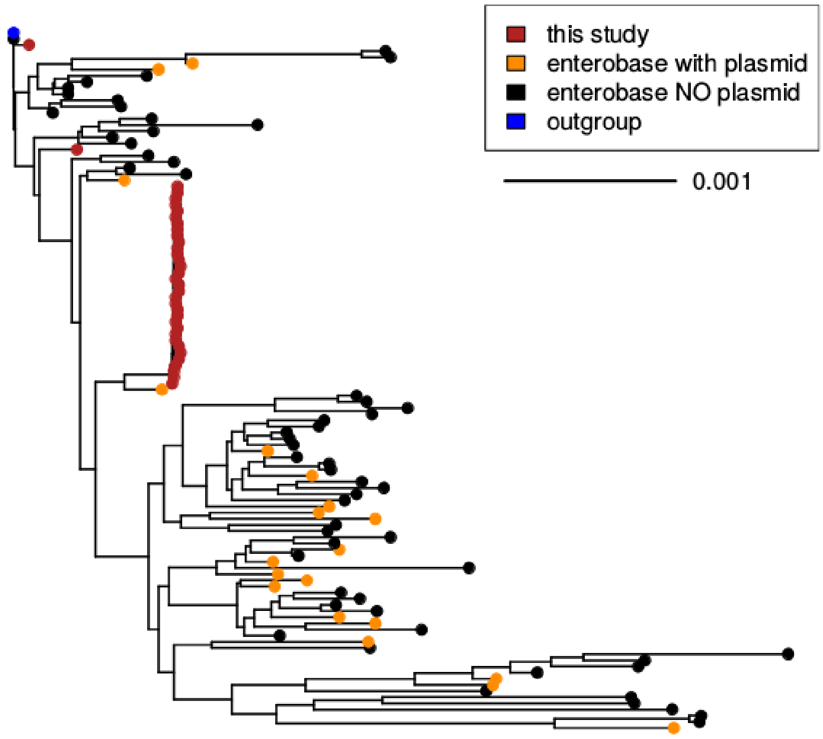

Figure S2. Phylogenetic tree of *E. coli* ST399. In orange and black the samples from EnteroBase, in orange the ones in which we found traces of the plasmids (although no complete plasmid was found in any of the samples) and in black the samples in which we did not find any trace of the plasmid, in blue the reference genome and in red the samples included in this study. Note that all except two of the samples from this study cluster exclusively into a single clade pointing to a clonal expansion of this specific clade.

Supplementary Table 1:

contains all the detailed information about each of the analised samples.

Species Identification

**Isolate**	**#1 Match**	**%**	**#2 Match**	**%**	**#3 Match**	**%**	**#4 Match**	**%**
cpe01	Escherichia coli	32.91	unclassified	7.55	Klebsiella oxytoca	1.01	Salmonella enterica	0.83
cpe02	Escherichia coli	33.28	unclassified	7.93	Klebsiella oxytoca	0.93	Salmonella enterica	0.83
cpe03	Escherichia coli	32.88	unclassified	8.02	Klebsiella oxytoca	1.02	Salmonella enterica	0.85
cpe04	Escherichia coli	32.58	unclassified	10.53	Klebsiella oxytoca	1.13	Salmonella enterica	0.8
cpe05	Escherichia coli	33.16	unclassified	7.62	Klebsiella oxytoca	1.07	Salmonella enterica	0.85
cpe06	Escherichia coli	32.71	unclassified	8.71	Klebsiella oxytoca	1.09	Salmonella enterica	0.84
cpe07	Escherichia coli	33.11	unclassified	8.23	Klebsiella oxytoca	0.93	Salmonella enterica	0.75
cpe08	Escherichia coli	32.97	unclassified	8.11	Klebsiella oxytoca	1	Salmonella enterica	0.83
cpe09	Escherichia coli	33.1	unclassified	8.53	Klebsiella oxytoca	1.01	Salmonella enterica	0.82
cpe10	Escherichia coli	33.02	unclassified	8.57	Klebsiella oxytoca	0.94	Salmonella enterica	0.82
cpe11	Escherichia coli	32.79	unclassified	8.13	Klebsiella oxytoca	1.03	Salmonella enterica	0.83
cpe12	Escherichia coli	35.07	unclassified	6.96	Klebsiella oxytoca	0.96	Salmonella enterica	0.82
cpe13	Escherichia coli	34.07	unclassified	6.54	Salmonella enterica	0.84	Shigella flexneri	0.63
cpe14	Escherichia coli	32.23	unclassified	9.56	Klebsiella oxytoca	0.95	Salmonella enterica	0.78

cpe15	Escherichia coli	34.07	unclassifie d	6.98	Salmonella enterica	0.85	Shigella flexneri	0.69
cpe16	Escherichia coli	33.3	unclassifie d	7.82	Klebsiella oxytoca	1	Salmonella enterica	0.77
cpe17	Escherichia coli	33.07	unclassifie d	8.29	Klebsiella oxytoca	1.03	Salmonella enterica	0.84
cpe18	Escherichia coli	32.74	unclassifie d	8.85	Klebsiella oxytoca	1.01	Shigella flexneri	0.6
cpe19	Escherichia coli	34	unclassifie d	7.01	Klebsiella oxytoca	1.08	Shigella flexneri	0.72
cpe21	Escherichia coli	70.4	unclassifie d	3.92	Salmonella enterica	1.24	Shigella boydii	0.56
cpe22	Escherichia coli	35.01	unclassifie d	6.53	Klebsiella oxytoca	0.93	Salmonella enterica	0.8
cpe23	Escherichia coli	36.04	unclassifie d	4.49	Enterobacter ia phage P1	0.34	Shigella dysenteriae	0.07
cpe24	unclassified	67.05	Citrobacter koseri	3.42	Salmonella enterica	2.99	Escherichia coli	2.17
cpe25	Klebsiella pneumoniae	26.91	Klebsiella variicola	18.49	unclassified	7.45	Salmonella enterica	0.57
cpe26	Klebsiella pneumoniae	27.83	Klebsiella variicola	18.23	unclassified	9.05	Enterobacter aerogenes	0.32
cpe27	Klebsiella pneumoniae	81.87	unclassifie d	8.87	Enterobacter cloacae	0.44	Escherichia coli	0.34
cpe28	Escherichia coli	75.74	unclassifie d	7.38	Escherichia fergusonii	0.15	Salmonella enterica	0.07
cpe30	Escherichia coli	33.19	unclassifie d	7.47	Klebsiella oxytoca	0.99	Salmonella enterica	0.83
cpe32	unclassified	67.02	Citrobacter koseri	3.39	Salmonella enterica	3.01	Escherichia coli	2.13
cpe33	Escherichia coli	31.68	unclassifie d	9.47	Salmonella enterica	2.01	Klebsiella oxytoca	1.03
cpe34	Escherichia coli	33.01	unclassifie d	8.15	Klebsiella oxytoca	1.09	Salmonella enterica	0.83
cpe35	Enterobacter cloacae	89.31	unclassifie d	7.3	Enterobacter asburiae	0.16	Klebsiella pneumoniae	0.08
cpe37	Escherichia	36.38	unclassifie	6.51	Enterobacter	0.41	Shigella	0.38

	coli		d		ia phage mEp237		boydii	
cpe38	Enterobacter cloacae	64.86	unclassifie d	13.67	Escherichia coli	5.89	Salmonella enterica	2.32
cpe39	Escherichia coli	30.71	unclassifie d	13.62	Klebsiella oxytoca	1.11	Salmonella enterica	0.82
cpe40	Escherichia coli	75.37	unclassifie d	3.92	Salmonella enterica	2.37	Cronobacter sakazakii	0.42
cpe41	Escherichia coli	32.92	unclassifie d	8.37	Klebsiella oxytoca	1.08	Salmonella enterica	0.83
cpe42	Klebsiella pneumoniae	81.93	unclassifie d	7.98	Klebsiella oxytoca	0.46	Escherichia coli	0.4
cpe43	Escherichia coli	32.93	unclassifie d	8.57	Klebsiella oxytoca	1.02	Salmonella enterica	0.87
cpe44	Enterobacter cloacae	72.52	unclassifie d	15.09	Klebsiella oxytoca	1.6	Salmonella enterica	0.57
cpe45	Escherichia coli	32.98	unclassifie d	8.18	Klebsiella oxytoca	1.13	Salmonella enterica	0.88
cpe46	Klebsiella pneumoniae	88.6	unclassifie d	5.84	Salmonella enterica	0.25	Klebsiella variicola	0.09
cpe47	Escherichia coli	32.79	unclassifie d	8.06	Klebsiella oxytoca	1.01	Salmonella enterica	0.83
cpe48	Klebsiella pneumoniae	88.84	unclassifie d	5.61	Klebsiella variicola	0.21	Enterobacter aerogenes	0.08
cpe50	Klebsiella pneumoniae	92.01	unclassifie d	3.45	Klebsiella variicola	0.15	Enterobacter aerogenes	0.05
cpe51	Escherichia coli	19.08	unclassifie d	7.91	Shigella boydii	1.04	Shigella sonnei	1.01
cpe52	Escherichia coli	33.07	unclassifie d	8.12	Klebsiella oxytoca	1.05	Salmonella enterica	0.87
cpe53	Escherichia coli	33.8	unclassifie d	6.97	Klebsiella oxytoca	1.08	Shigella flexneri	0.62
cpe54	Escherichia coli	33.39	unclassifie d	6.89	Klebsiella oxytoca	1.11	Salmonella enterica	0.87
cpe55	Escherichia coli	33.29	unclassifie d	7.49	Klebsiella oxytoca	1	Salmonella enterica	0.83
cpe56	Escherichia	62.34	unclassifie	4.91	Escherichia	0.71	Salmonella	0.44

	coli		d		fergusonii		enterica	
cpe57	Escherichia coli	33.38	unclassified	7.6	Klebsiella oxytoca	1.01	Salmonella enterica	0.76
cpe58	Escherichia coli	32.58	unclassified	9.03	Klebsiella oxytoca	1.11	Salmonella enterica	0.87
cpe59	Escherichia coli	41.86	unclassified	4.39	Klebsiella oxytoca	1.06	Yersinia pestis	0.5
cpe60	Escherichia coli	33.34	unclassified	7.73	Klebsiella oxytoca	1.08	Salmonella enterica	0.9

mlst

Isolate	Scheme	Sequence Type	Allele	Allele	Allele	Allele	Allele	Allele	Allele
cpe01	ecoli	399	adk(6)	fumC(4)	gyrB(1)	icd(95)	mdh(69)	purA(8)	recA(20)
cpe02	ecoli	399	adk(6)	fumC(4)	gyrB(1)	icd(95)	mdh(69)	purA(8)	recA(20)
cpe03	ecoli	399	adk(6)	fumC(4)	gyrB(1)	icd(95)	mdh(69)	purA(8)	recA(20)
cpe04	ecoli	399	adk(6)	fumC(4)	gyrB(1)	icd(95)	mdh(69)	purA(8)	recA(20)
cpe05	ecoli	399	adk(6)	fumC(4)	gyrB(1)	icd(95)	mdh(69)	purA(8)	recA(20)
cpe06	ecoli	399	adk(6)	fumC(4)	gyrB(1)	icd(95)	mdh(69)	purA(8)	recA(20)
cpe07	ecoli	399	adk(6)	fumC(4)	gyrB(1)	icd(95)	mdh(69)	purA(8)	recA(20)
cpe08	ecoli	399	adk(6)	fumC(4)	gyrB(1)	icd(95)	mdh(69)	purA(8)	recA(20)
cpe09	ecoli	399	adk(6)	fumC(4)	gyrB(1)	icd(95)	mdh(69)	purA(8)	recA(20)
cpe10	ecoli	399	adk(6)	fumC(4)	gyrB(1)	icd(95)	mdh(69)	purA(8)	recA(20)
cpe11	ecoli	399	adk(6)	fumC(4)	gyrB(1)	icd(95)	mdh(69)	purA(8)	recA(20)
cpe12	ecoli	399	adk(6)	fumC(4)	gyrB(1)	icd(95)	mdh(69)	purA(8)	recA(20)
cpe13	ecoli	399	adk(6)	fumC(4)	gyrB(1)	icd(95)	mdh(69)	purA(8)	recA(20)
cpe14	ecoli	399	adk(6)	fumC(4)	gyrB(1)	icd(95)	mdh(69)	purA(8)	recA(20)
cpe15	ecoli	399	adk(6)	fumC(4)	gyrB(1)	icd(95)	mdh(69)	purA(8)	recA(20)
cpe16	ecoli	399	adk(6)	fumC(4)	gyrB(1)	icd(95)	mdh(69)	purA(8)	recA(20)

cpe17	ecoli	399	adk(6)	fumC(4)	gyrB(1)	icd(95)	mdh(69)	purA(8)	recA(20)
cpe18	ecoli	399	adk(6)	fumC(4)	gyrB(1)	icd(95)	mdh(69)	purA(8)	recA(20)
cpe19	ecoli	399	adk(6)	fumC(4)	gyrB(1)	icd(95)	mdh(69)	purA(8)	recA(20)
cpe21	ecoli	69	adk(21)	fumC(35)	gyrB(27)	icd(6)	mdh(5)	purA(5)	recA(4)
cpe22	ecoli	399	adk(6)	fumC(4)	gyrB(1)	icd(95)	mdh(69)	purA(8)	recA(20)
cpe23	ecoli	-	adk(6)	fumC(6)	gyrB(5)	icd(1)	mdh(9)	purA(7)	recA(7)
cpe24	ecloacae	-	dnaA(~110)	fusA(~103)	gyrB(~121)	leuS(~128)	pyrG(-)	rplB(~52)	rpoB(70)
cpe25	kpneumoniae	-	gapA(16)	infB(28)	mdh(~30)	pgi(49)	phoE(83)	rpoB(22)	tonB(124)
cpe26	kpneumoniae	1169	gapA(16)	infB(24)	mdh(21)	pgi(27)	phoE(47)	rpoB(22)	tonB(206)
cpe27	kpneumoniae	11	gapA(3)	infB(3)	mdh(1)	pgi(1)	phoE(1)	rpoB(1)	tonB(4)
cpe28	ecoli	-	adk(13)	fumC(43)	gyrB(19)	icd(~37)	mdh(17)	purA(25)	recA(25)
cpe30	ecoli	399	adk(6)	fumC(4)	gyrB(1)	icd(95)	mdh(69)	purA(8)	recA(20)
cpe32	senterica	-	aroC(-)	dnaN(167?)	hemD(~316)	hisD(-)	purE(-)	sucA(-)	thrA(-)
cpe33	ecoli	399	adk(6)	fumC(4)	gyrB(1)	icd(95)	mdh(69)	purA(8)	recA(20)
cpe34	ecoli	399	adk(6)	fumC(4)	gyrB(1)	icd(95)	mdh(69)	purA(8)	recA(20)
cpe35	ecloacae	-	dnaA(4)	fusA(~4)	gyrB(4)	leuS(~6)	pyrG(39)	rplB(4)	rpoB(25)
cpe37	ecoli	4995	adk(6)	fumC(4)	gyrB(5)	icd(1)	mdh(8)	purA(8)	recA(6)
cpe38	ecloacae	144	dnaA(74)	fusA(20)	gyrB(74)	leuS(78)	pyrG(45)	rplB(35)	rpoB(32)
cpe39	ecoli	399	adk(6)	fumC(4)	gyrB(1)	icd(95)	mdh(69)	purA(8)	recA(20)
cpe40	ecoli	131	adk(53)	fumC(40)	gyrB(47)	icd(13)	mdh(36)	purA(28)	recA(29)
cpe41	ecoli	399	adk(6)	fumC(4)	gyrB(1)	icd(95)	mdh(69)	purA(8)	recA(20)
cpe42	kpneumoniae	788	gapA(2)	infB(4)	mdh(2)	pgi(1)	phoE(7)	rpoB(1)	tonB(12)
cpe43	ecoli	399	adk(6)	fumC(4)	gyrB(1)	icd(95)	mdh(69)	purA(8)	recA(20)
cpe44	ecloacae	182	dnaA(49)	fusA(20)	gyrB(19)	leuS(44)	pyrG(90)	rplB(24)	rpoB(32)
cpe45	ecoli	399	adk(6)	fumC(4)	gyrB(1)	icd(95)	mdh(69)	purA(8)	recA(20)
cpe46	kpneumoniae	-	gapA(2)	infB(1)	mdh(1)	pgi(26)	phoE(10)	rpoB(1)	tonB(15)
cpe47	ecoli	399	adk(6)	fumC(4)	gyrB(1)	icd(95)	mdh(69)	purA(8)	recA(20)

cpe48	kpneumoniae	1537	gapA(2)	infB(1)	mdh(2)	pgi(1)	phoE(12)	rpoB(1)	tonB(46)
cpe50	kpneumoniae	-	gapA(3)	infB(1)	mdh(6)	pgi(36)	phoE(12)	rpoB(4)	tonB(38)
cpe51	ecoli	3168	adk(1)	fumC(23)	gyrB(7)	icd(11)	mdh(7)	purA(3)	recA(7)
cpe52	ecoli	399	adk(6)	fumC(4)	gyrB(1)	icd(95)	mdh(69)	purA(8)	recA(20)
cpe53	ecoli	399	adk(6)	fumC(4)	gyrB(1)	icd(95)	mdh(69)	purA(8)	recA(20)
cpe54	ecoli	399	adk(6)	fumC(4)	gyrB(1)	icd(95)	mdh(69)	purA(8)	recA(20)
cpe55	ecoli	399	adk(6)	fumC(4)	gyrB(1)	icd(95)	mdh(69)	purA(8)	recA(20)
cpe56	ecoli	349	adk(34)	fumC(36)	gyrB(39)	icd(87)	mdh(67)	purA(16)	recA(4)
cpe57	ecoli	399	adk(6)	fumC(4)	gyrB(1)	icd(95)	mdh(69)	purA(8)	recA(20)
cpe58	ecoli	399	adk(6)	fumC(4)	gyrB(1)	icd(95)	mdh(69)	purA(8)	recA(20)
cpe59	ecoli	10	adk(10)	fumC(11)	gyrB(4)	icd(8)	mdh(8)	purA(8)	recA(2)
cpe60	ecoli	399	adk(6)	fumC(4)	gyrB(1)	icd(95)	mdh(69)	purA(8)	recA(20)

Assembly

Isolate	Contigs	bp	ok	Ns	gaps	min	avg	max	N50	Insert size (25~50~75)%	CDS	rRNA	tRNA	tmRNA
cpe01	224	5167721	5167721	0	0	502	23070	259267	82081	(313~392~493)	4940	9	79	
cpe02	213	5163527	5163527	0	0	502	24241	278585	84021	(315~394~490)	4936	10	81	1
cpe03	284	5227654	5227654	0	0	500	18407	259267	83450	(304~370~454)	4982	10	78	1
cpe04	222	5141048	5141048	0	0	502	23157	259267	82081	(304~377~471)	4907	10	79	1
cpe05	224	5165172	5165172	0	0	502	23058	278585	82081	(302~367~447)	4933	10	82	1
cpe06	211	5139501	5139501	0	0	502	24357	278585	85739	(287~	4905	9	78	1

										349~ 427)				
cpe07	220	5115732	5115732	0	0	502	23253	278585	81669	(312~ 383~ 473)	4890	9	86	1
cpe08	221	5168595	5168595	0	0	502	23387	259267	82081	(328~ 409~ 506)	4939	8	79	1
cpe09	222	5170598	5170598	0	0	502	23290	278585	85920	(317~ 388~ 475)	4938	10	80	1
cpe10	221	5168431	5168431	0	0	502	23386	259267	82081	(299~ 374~ 466)	4938	9	77	1
cpe11	224	5167624	5167624	0	0	502	23069	278585	84096	(301~ 372~ 461)	4935	8	76	1
cpe12	218	5255776	5255776	0	0	500	24109	259267	81669	(325~ 405~ 502)	5034	9	78	1
cpe13	205	5093896	5093896	0	0	502	24848	278585	87191	(293~ 359~ 442)	4853	9	80	1
cpe14	195	5010805	5010805	0	0	508	25696	259075	86582	(295~ 362~ 446)	4824	9	78	1
cpe15	213	5093356	5093356	0	0	502	23912	278585	82081	(308~ 377~ 467)	4851	9	78	1
cpe16	208	5091494	5091494	0	0	502	24478	278585	84096	(301~ 367~ 450)	4859	9	79	1
cpe17	222	5168125	5168125	0	0	502	23279	259267	82081	(302~ 368~ 452)	4938	9	77	1
cpe18	211	5077742	5077742	0	0	502	24065	278585	87225	(323~ 406~ 509)	4843	9	80	1
cpe19	212	5095545	5095545	0	0	502	24035	278585	87225	(306~ 374~ 460)	4850	9	80	1
cpe21	230	5419582	5419582	0	0	500	23563	264094	101348	(300~ 376~ 469)	5195	5	86	1

cpe22	275	5298504	5298504	0	0	502	19267	202887	68627	(332~ 403~ 490)	5072	10	80	1
cpe23	104	4800971	4800971	0	0	518	46163	405502	166216	(302~ 369~ 451)	4506	7	80	1
cpe24	45	4987539	4987539	0	0	523	110834	1343191	445774	(282~ 361~ 459)	4672	4	76	1
cpe25	68	5599335	5599335	0	0	513	82343	484926	257294	(260~ 332~ 425)	5214	5	78	1
cpe26	71	5987493	5987493	0	0	523	84330	549695	192943	(282~ 367~ 467)	5615	5	80	1
cpe27	105	5567084	5567084	0	0	501	53019	432720	143062	(221~ 287~ 364)	5271	8	78	1
cpe28	55	4686326	4686326	0	0	507	85205	537249	278633	(309~ 382~ 473)	4312	9	77	1
cpe30	222	5168904	5168904	0	0	502	23283	278585	82081	(306~ 373~ 455)	4936	10	79	1
cpe32	48	4990276	4990276	0	0	523	103964	753286	523696	(234~ 308~ 397)	4671	3	75	1
cpe33	230	5261504	5261504	0	0	502	22876	259075	83450	(299~ 370~ 460)	5046	9	78	1
cpe34	209	5143520	5143520	0	0	502	24610	278585	84096	(290~ 355~ 436)	4915	9	78	1
cpe35	45	4669241	4669241	0	0	528	103760	589472	353910	(277~ 361~ 468)	4326	1	72	1
cpe37	93	4965454	4965454	0	0	507	53391	399456	172365	(302~ 369~ 456)	4669	6	76	1
cpe38	114	5067327	5067327	0	0	506	44450	435723	146719	(254~ 329~ 421)	4827	7	80	1
cpe39	223	5168483	5168483	0	0	502	23177	278585	83370	(312~ 380~ 466)	4944	9	78	1

cpe40	179	5394704	5394704	0	0	501	30138	409918	127990	(309~ 373~ 456)	5086	8	75	1
cpe41	219	5171253	5171253	0	0	502	23613	259076	83268	(307~ 378~ 470)	4944	10	80	1
cpe42	133	5689405	5689405	0	0	504	42777	351373	180432	(234~ 302~ 393)	5307	3	79	1
cpe43	218	5167345	5167345	0	0	502	23703	278585	81669	(309~ 386~ 481)	4942	9	78	1
cpe44	108	5187152	5187152	0	0	516	48029	406097	129153	(263~ 340~ 431)	4945	3	78	1
cpe45	219	5161686	5161686	0	0	502	23569	278585	84607	(286~ 350~ 430)	4926	10	80	1
cpe46	80	5146402	5146402	0	0	513	64330	560849	203046	(248~ 318~ 406)	4744	4	78	1
cpe47	223	5163374	5163374	0	0	502	23154	197351	82081	(303~ 371~ 458)	4935	9	75	1
cpe48	57	5162309	5162309	0	0	577	90566	509005	226349	(244~ 309~ 388)	4802	6	78	1
cpe50	70	5238721	5238721	0	0	540	74838	456946	208322	(235~ 301~ 385)	4856	5	76	1
cpe51	220	4931400	4931400	0	0	509	22415	187042	68038	(310~ 380~ 464)	4700	6	74	1
cpe52	217	5168800	5168800	0	0	502	23819	259267	83450	(304~ 376~ 467)	4944	10	79	1
cpe53	208	5097049	5097049	0	0	502	24505	278585	86582	(299~ 364~ 447)	4855	9	81	1
cpe54	213	5163229	5163229	0	0	502	24240	278585	84096	(290~ 349~ 427)	4938	8	77	1
cpe55	217	5169965	5169965	0	0	502	23824	278585	83226	(306~ 373~ 459)	4943	9	79	1

cpe56	153	5039877	5039877	0	0	500	32940	357663	127835	(304~ 375~ 465)	4685	5	84	1
cpe57	208	5108571	5108571	0	0	502	24560	258965	85739	(309~ 385~ 483)	4876	10	79	1
cpe58	224	5168496	5168496	0	0	502	23073	278585	82081	(289~ 358~ 445)	4939	9	79	1
cpe59	97	4778718	4778718	0	0	500	49265	396320	154361	(287~ 358~ 449)	4483	5	76	1
cpe60	219	5165072	5165072	0	0	502	23584	278585	82081	(310~ 384~ 475)	4936	9	77	1

Sequencing Data

Isolate	Reads	Yield	GC	MinLen	AvgLen	MaxLen	ModeLen	Phred	AvgQual	Depth
cpe01	3282524	491743914	50.5	30	149	150	150	33	36.1	93
cpe02	3101672	464695660	50.2	30	149	150	150	33	36.5	88
cpe03	3160426	473537507	50.9	30	149	150	150	33	36.8	90
cpe04	3359828	503392446	50	30	149	150	150	33	36.7	96
cpe05	3565542	534259959	51	30	149	150	150	33	36.6	101
cpe06	4044806	606092447	50.5	30	149	150	150	33	36.9	115
cpe07	3352210	502279957	50.5	30	149	150	150	33	36.7	95
cpe08	3273398	490397845	50.6	30	149	150	150	33	36.2	93
cpe09	3364210	504049643	50.4	30	149	150	150	33	36.6	96
cpe10	3535980	529783264	50.3	30	149	150	150	33	36.7	101
cpe11	3295834	493802301	50.8	30	149	150	150	33	36.6	94
cpe12	2942776	440865812	50.8	30	149	150	150	33	36.6	84
cpe13	3550170	531955315	51.1	30	149	150	150	33	36.9	101
cpe14	3531296	529121961	50.3	30	149	150	150	33	36.7	100
cpe15	3088482	462742054	50.9	30	149	150	150	33	36.6	88

cpe16	3842338	575709420	50.8	30	149	150	150	33	36.8	109
cpe17	3831186	574038292	50.7	30	149	150	150	33	37	109
cpe18	2787348	417543019	51.4	30	149	150	150	33	36.2	79
cpe19	3239484	485379244	50.7	30	149	150	150	33	36.8	92
cpe21	3362334	503709024	50.5	30	149	150	150	33	36.5	96
cpe22	3432848	514287867	52	30	149	150	150	33	36.3	98
cpe23	3662380	548726944	51.4	30	149	150	150	33	36.6	104
cpe24	3344286	501070743	51.2	30	149	150	150	33	36.6	95
cpe25	3251388	486899642	56.3	30	149	150	150	33	35.9	92
cpe26	2609138	390615161	56.9	30	149	150	150	33	35.5	74
cpe27	3696294	553595543	56.1	30	149	150	150	33	37	105
cpe28	3426606	513427322	50.6	30	149	150	150	33	36.9	97
cpe30	3851594	577075742	51.3	30	149	150	150	33	36.6	110
cpe32	3768938	564678666	51.3	30	149	150	150	33	37.1	107
cpe33	3565796	534264956	50.3	30	149	150	150	33	36.8	101
cpe34	3648258	546602769	51.3	30	149	150	150	33	36.8	104
cpe35	2835770	424709781	55.5	30	149	150	150	33	36	81
cpe37	3452288	517252187	50.5	30	149	150	150	33	36.8	98
cpe38	3778064	565956473	54.6	30	149	150	150	33	36.7	108
cpe39	4046544	606267357	51.6	30	149	150	150	33	36.6	115
cpe40	4154442	622464608	50.8	30	149	150	150	33	36.8	118
cpe41	3425736	513249503	50.5	30	149	150	150	33	36.7	97
cpe42	4010596	600715911	56.2	30	149	150	150	33	36.9	114
cpe43	3235084	484654238	50.7	30	149	150	150	33	36.4	92
cpe44	3105122	465143526	55.7	30	149	150	150	33	36.5	88
cpe45	3617974	542066148	50.9	30	149	150	150	33	37	103
cpe46	3606642	540118708	57.3	30	149	150	150	33	36.3	103
cpe47	3891468	583024183	51.1	30	149	150	150	33	36.7	111
cpe48	3765992	564037567	57	30	149	150	150	33	36.7	107
cpe50	3874770	580291799	58.5	30	149	150	150	33	36.6	110
cpe51	3406564	510325927	53.2	30	149	150	150	33	36.5	97

cpe52	3466408	519359117	51	30	149	150	150	33	36.8	99
cpe53	3632022	544191421	51.4	30	149	150	150	33	36.8	103
cpe54	3936074	589753248	51.7	30	149	150	150	33	37	112
cpe55	3096746	464000064	51	30	149	150	150	33	36.8	88
cpe56	3639020	545242122	50.4	30	149	150	150	33	36.9	104
cpe57	3014492	451637096	50.7	30	149	150	150	33	36.5	86
cpe58	3251054	487045408	50.7	30	149	150	150	33	36.6	92
cpe59	3492112	523130505	51.1	30	149	150	150	33	36.8	99
cpe60	3548478	531642581	51.1	30	149	150	150	33	36.7	101

Epidemiological Data

Isolation Date	Sample ID	Patient ID	Isolation site	Specimen Date	Becterial ID	Resistance ID
May-16	cpe18	1	Rectal Screen	23/05/2016	E.coli	Oxa 48
Jun-16	cpe46	3	Rectal screen	13/06/2016	Kleb pneumo	Oxa 48
Jul-16	cpe19	5	Screen	07/07/2016	Oxa 48	OXA-48 28/7/16
Jul-16	cpe44	7	Rectal Screen	19/07/2016	Enterobacter cloacae	Oxa 48
Jul-16	cpe07	8	Groin	26/07/2016	OXA-48	OXA-48 4/8/16
Jul-16	cpe13	9	Rectal Screen	26/07/2016	E.coli	OXA-48
Aug-16	cpe14	10	Rectal	02/08/2016	E.coli	OXA-48
Aug-16	cpe42	11	Rectal Screen	11/08/2016	E.coli/Kleb pneumo	OXA-48
Aug-16	cpe40	11	Rectal Screen	11/08/2016	E.coli/Kleb pneumo	OXA-48
Aug-16	cpe45	13	Rectal Screen	10/08/2016	E.coli	OXA-48
Aug-16	cpe15	14	Rectal	22/08/2016	E.coli	OXA-48
Sep-16	cpe23	15	Rectal	23/08/2016	E.coli/Enterobacter cloacae	OXA-48
Sep-16	cpe35	15	Rectal	23/08/2016	E.coli/Enterobacter cloacae	OXA-48

Sep-16	cpe21	17	Rectal	19/09/2016	E.coli	Oxa 48
Sep-16	cpe22	19	Rectal	26/09/2016	E.coli	OXA-48
Oct-16	cpe08	20	Rectal	26/09/2016	E.coli	OXA-48
Oct-16	cpe09	21	Rectal	26/09/2016	E.coli	OXA-48
Oct-16	cpe30	21	Rectal	26/09/2016	E.coli	OXA-48
Oct-16	cpe47	25	Screen	03/10/2016	E.colix3	OXA-48
Oct-16	cpe10	26	Rectal Screen	10/10/2016	E.coli	OXA-48
Nov-16	cpe24	27	Rectal	18/10/2016	E.coli	OXA-48
Nov-16	cpe32	28	Rectal	18/10/2016	E.coli- Citrobacter freundii	OXA-48
Nov-16	cpe37	28	Rectal	18/10/2016	E.coli- Citrobacter freundii	OXA-48
Nov-16	cpe11	31	Rectal	31/10/2016	E.coli	OXA-48
Nov-16	cpe17	34	Rectal	01/11/2016	E.coli	OXA-48
Nov-16	cpe03	32	Rectal	01/11/2016	E.coli	OXA-48
Nov-16	cpe34	33	Rectal	01/11/2016	E.coli	OXA-48
Nov-16	cpe04	35	Screen	01/11/2016	E.ccoli	OXA-48
Nov-16	cpe48	36	Rectal	07/11/2016	Kleb neumo (2 isolates)	OXA-48
Nov-16	cpe12	37	Rectal	07/11/2016	E.coli	OXA-48
Nov-16	cpe16	38	Rectal	07/11/2016	K.pneumo- E.coli	OXA-48
Nov-16	cpe50	40	Rectal	07/11/2016	Kleb pneumo- E.coli	OXA-48
Nov-16	cpe43	44	Rectal	14/11/2016	E.coli	OXA-48
Nov-16	cpe41	42	Rectal	14/11/2016	E.coli	OXA-48
Nov-16	cpe02	41	Rectal	14/11/2016	E.coli	0XA-48
Nov-16	cpe05	52	Rectal	22/11/2016	1) E.coli 2) Kleb pneumo	OXA-48
Nov-16	cpe06	54	rectal	21/11/2016	E.coli	OXA-48
Nov-16	cpe33	53	Rectal	21/11/2016	1) E.coli- 2) Ent. Cloacae	OXA-48
Nov-16	cpe38	53	Rectal	21/11/2016	1) E.coli	OXA-48

Jan-17	cpe26	51	Rectal	21/11/2016	Kleb pneumo	OXA-48
Jan-17	cpe25	56	Rectal	21/11/2016	1) E.coli 2) Kleb pneumo	OXA-48
Jan-17	cpe28	56	Rectal	21/11/2016	1) E.coli	OXA-48
Jan-17	cpe27	55	Rectal	21/11/2016	Kleb pneumo	OXA-48
Jan-17	cpe39	57	Rectal	21/11/2016	E.coli	OXA-48
Jan-17	cpe01	58	Rectal	21/11/2016	E.coli	OXA-48
Jan-17	cpe51	61	rectal	29/11/16	E.coli	OXA-48
Jan-17	cpe53	73	screen	03/01/2017	E.coli	OXA-48
Jan-17	cpe52	70	rectal	03/01/2017	E.coli x2	OXA-48
Jan-17	cpe54	76	faeces	09/01/2017	E.coli	OXA-48
Jan-17	cpe59	93	screen	06/02/2017	E.coli	OXA-48
Feb-17	cpe55	78	faeces/rectum	16/01/2017	E.coli	OXA-48
Feb-17	cpe56	82	Rectal	16/01/2017	OXA-48	OXA48 27/1/17
Feb-17	cpe57	85	Rectal	22/01/2017	E.coli	OXA-48
Feb-17	cpe60	94	screen	06/02/2017	E.coli	OXA-48
